# Quercetin Increases Growth Performance and Decreases Incidence of Diarrhea and Mechanism of Action in Weaned Piglets

**DOI:** 10.1155/2024/5632260

**Published:** 2024-08-06

**Authors:** Yanjun Mao, Qinglin Yang, Junhong Liu, Yuxin Fu, Shuaishuai Zhou, Jiayan Liu, Linlin Ying, Yao Li

**Affiliations:** College of Animal Science and Technology Northeast Agricultural University, Harbin 150030, China

## Abstract

This study aimed to investigate the mechanism of quercetin increasing growth performance and decreasing incidence of diarrhea in weaned piglets. Forty-eight Duroc × Landrace × Large White weaned piglets with similar body weight (7.48 ± 0.20 kg, 28 days of age) were randomly divided into four treatments (control, 250 mg/kg quercetin, 500 mg/kg quercetin, and 750 mg/kg quercetin treatments) and fed with basal diet or experimental diet supplemented with quercetin. Performance, diarrhea rate and index, and content of serum anti-inflammatory factors were determined and calculated in weaned piglets; colonic flora and signaling pathways related to anti-inflammation were measured using 16S rDNA sequencing and RNA-seq, respectively. The results showed that compared with control, feed-to-gain ratio and content of serum interferon gamma (IFN-*γ*) were significantly decreased in the 500 and 750 mg/kg quercetin treatments (*P* < 0.05); quercetin significantly decreased diarrhea rate and diarrhea index (*P* < 0.05) and significantly increased the content of serum transforming growth factor (TGF-*β*) in weaned piglets (*P* < 0.05); the content of serum NF-*κ*B was significantly decreased in the 750 mg/kg quercetin treatment (*P* < 0.05); moreover, quercetin significantly increased diversity of colonic flora (*P* < 0.05), and at the phylum level, the relative abundance of Actinobacteria in the 500 and 750 mg/kg treatments was significantly increased (*P* < 0.05), and the relative abundance of Proteobacteria in the three quercetin treatments were significantly decreased (*P* < 0.05) in the colon of weaned piglets; at the genus level, the relative abundance of *Clostridium-sensu-stricto-1*, *Turicibacter*, *unclassified_f_Lachnospiraceae*, *Phascolarctobacterium*, and *Family_XIII _AD3011_group* was significantly increased (*P* < 0.05); the relative abundance of *Subdollgranulum* and *Blautia* was significantly decreased in the 500 and 750 mg/kg treatments (*P* < 0.05); the relative abundance of *Eschericha-Shigella*, *Terrisporobacter*, and *Eubacterium-coprostanoligenes* was significantly increased (*P* < 0.05); the relative abundance of *Streptocococcus*, *Sarcina*, *Staphylococcus*, and *Ruminococcaceae_UCG-008* was significantly decreased in the three quercetin treatments (*P* < 0.05); the relative abundance of *Ruminococcaceae_UCG_014* was significantly increased in the 250 mg/kg quercetin treatment in the colon of weaned piglets (*P* < 0.05). The results of Gene Ontology (GO) analysis and Kyoto Encyclopedia of Genes and Genomes (KEGG) pathway analysis showed that differentially expressed genes (DEGs) from the quercetin treatments were significantly enriched in nuclear transcription factor-*κ*B (NF-*κ*B) signal pathway (*P* < 0.05); mRNA expression of tumor necrosis factor-*α* (TNF-*α*), interleukin-1R1 (IL-1R1), conserved helix-loop-helix ubiquitous kinase (CHUK), toll-like receptor 4 (TLR4), and IL-1*β* from quercetin treatments were significantly decreased in colonic mucosa of weaned piglets (*P* < 0.05). In summary, quercetin increased feed conversion ratio and decreased diarrhea through regulating NF-*κ*B signaling pathway, controlling the balance between anti-inflammatory and proinflammatory factors, and modulating intestinal flora, thus promoting the absorption of nutrients in weaned piglets. These results provided the theoretical foundation for applying quercetin in preventing weaning piglets' diarrhea and animal husbandry practices.

## 1. Introduction

The health of piglets is affected by environment, nutrition and feed replacement, etc. However, the intestinal health of piglets was greatly challenged by changing postweaned factors [1, 2]. The transformation from liquid milk to solid feed may damage intestinal villi, intestinal absorption, and secretion of digestive enzymes, which reduced growth rate, prolonged hunger period, and severe weaning anorexia [3, 4]. Lack of immune proteins from breast milk and poor development of the immune system may trigger a series of inflammatory responses, thus contributing to postweaning diarrhea [5]. High cost of resolving the above-mentioned problems, poor growth, and high mortality may bring about huge economic losses. Antibiotic additives may prevent these symptoms or shorten the duration of these symptoms and, however, increase antibiotic resistances in intestinal microorganisms to antibiotics and growth promoters; thereby, antibiotic additives have been prohibited, and antibiotic alternatives are imperative to develop [6]. Over the decades, the beneficial effects of flavonoid-containing plant extracts have caught researchers' eyes in antibiotic substitution. Flavonoids are widely distributed in plants and have a variety of biological activities, including antibacterial, immune-regulatory, antioxidative, and anti-inflammatory capacities [7, 8]. Forty milligram per kilogram of tartary buckwheat flavonoids increased growth performance, antioxidative capacity, and immune function in 50 weaned piglets at 35 days of age [9]. New plant extracts (green tea and pomegranate peel) improved intestinal health and microecological balance in 144 weaned piglets at 24 days of age for 35 days [10]. Resveratrol and curcumin significantly modulated intestinal microflora, downregulated the Toll-like receptor 4 (TLR4) signaling pathway, reduced intestinal inflammation, and thus improved intestinal immune function in 180 weaned piglets at 28 days of age for 28 days [11]. Flavonoid aglycones effectively inhibited signaling pathways involved in inflammatory processes, including mitogen-activated protein kinases' (MAPKs) phosphorylation, inhibitor of *κ* B kinase (IKK) *α*/*β*, c-Jun N-terminal kinase (c-Jun), cyclic adenosine monophosphate (cAMP) response element binding protein, activated transcription factor 2, and nuclear factor *κ*BP65 [12]. Therefore, flavonoids have a broad development prospect in regulating antioxidation and immune function and improving growth performance as feed additives for animals.

Quercetin is one of the typical representatives of flavonoids, which are also abundant in onions and apples [13]. It exists in the form of glycosides in vegetables and fruits and exhibits rich biological activity [14]. Quercetin promoted the proliferation of porcine intestinal epithelial cells [15]. It also inhibited the growth of *Bifidobacterium catenulatum* and *Enterococcus cacca* and indirectly regulated the expression of genes responsible for protein folding, purine synthesis, and metabolism [16]. Moreover, 30 mg/kg quercetin directly stimulated immune system and maintained health through reducing inflammation and enhancing populations of *Bacteroides*, *Bifidobacterium*, *Lactobacillus*, and *Clostridia* in mice [17]. Quercetin inhibited TNF-*α* induced apoptosis and inflammatory response by blocking NF-*κ*B and activator protein-1 (AP-1) signaling pathway in human umbilical vein endothelial cells (HUVECs) [18]. In addition, quercetin ameliorated inflammation by regulating expression of NF-*κ*B, cyclooxygenase-2 (COX-2) protein, and NO in vitro [19].

The liver, intestine, and kidney are mainly involved in metabolism and excretion of quercetin [20]. The content of quercetin aglycone in colon was three times higher than that in the jejunum; thus, the colon was the main accumulation site of quercetin [21]. Most of the researches on quercetin focused on poultry, mice, and humans and has achieved remarkable results as a drug or additive to regulate immune, antioxidation, and intestinal flora balance. However, there are few reports on quercetin as a feed additive in pigs for antibiotic substitution and especially on detecting specific intestinal segments of weaned piglets after quercetin treatment. The bacteriostatic and anti-inflammatory mechanism of quercetin remained unclear in weaned piglets. Therefore, in this study, transcriptome sequencing and 16S rDNA sequencing were used to clarify the bacteriostatic and anti-inflammatory effect of quercetin and mechanism of action in weaned piglets; this will provide scientific basis for decreasing diarrhea in weaned piglets and applying quercetin in pig production.

## 2. Materials and Methods

The feeding experiment was carried out at Dingfeng Pig Farm in Taian, Liaoning Province, China. The quercetin was purchased from Nanjing Dulai Biotechnology Co., Ltd., and the purity of the sample was 95% (high-performance liquid chromatography (HPLC)).

### 2.1. Animals, Diet, and Management

Forty-eight Duroc × Landrace × Large White weaned piglets with similar body weight (7.48 ± 0.20 kg, 28 days of age) were randomly divided into four treatments with six replicates each treatment and separately housed; there were two piglets in each replicate. Piglets in control, three quercetin treatments were fed by basal diet supplemented with 0, 250, 500, and 750 mg/kg quercetin, respectively. The basal diet was formulated in accordance with the NCR (2012) standard and met the nutritional needs of weaned piglets without antibiotics and other additives (see Table 1). During the experiment, piglets were given access to feed and water *ad libitum*. The experiment lasted 35 days. The body weight of weaned piglets was recorded at the beginning and end of the experiment for calculating average daily feed intake (ADFI), average daily gain (ADG), and feed-to-gain ratio. Meanwhile, the diarrhea of piglets was observed and recorded every day, and diarrhea rate and diarrhea index were calculated. The scoring criteria for diarrhea are as follows: (1) feces are normal, soft, and shaped, normal; (2) feces are loose, partially formed, and mild diarrhea; and (3) semiliquid feces, feces and water are not separated, and moderate diarrhea [20].

### 2.2. Sample Collection

At the end of the experiment, one of the piglets was randomly selected from each replicate, and the blood was collected from the anterior vena cava with vacuum centrifuge tube. Blood samples were centrifuged for 10 min at 3,500 rpm for collecting serum and stored at −20°C for further analysis. Meanwhile, the colonic contents from slaughtered piglets after blood collection were collected under aseptic condition and quickly frozen in liquid nitrogen and stored at −80°C. Then the colon was rinsed with saline and mucosa of the colon was scraped, and immediately frozen in liquid nitrogen, and stored at −80°C for subsequent analysis. All procedures used in this study were approved by the Animal Care and Use Committee of the University (NEAUEC20200203). Housing, management, and care of the pigs confirmed to the guidelines of Agricultural Animal in Agricultural Research and Teaching of Heilongjiang Province (HEI Animal Management Certificate No. 11928).

### 2.3. Serum Index Determination

Content of TNF-*α*, IFN-*γ*, NF-*κ*B, and transforming growth factor (TGF-*β*) in serum of piglets was determined using ELISA kit purchased from Jiangsu Baolai Biotechnology Co., Ltd. (Jiangsu, China). The parameters were determined and analyzed according to the kit instructions.

### 2.4. Analysis of Microbial Diversity in the Colon

Microbial community genomic DNA was extracted from colonic contents using the commercial QIAamp DNA Stool Mini Kit (Omega Bio-tek, Norcross, GA, USA) according to the manufacturer's instructions. The DNA extract was checked on 1% agarose gel, and DNA concentration and purity were determined with NanoDrop 2,000 UV-vis spectrophotometer (Thermo Scientific, Wilmington, USA). The hypervariable region V3–V4 of the bacterial 16S rDNA gene was amplified with primer pairs 338F (5′-ACTCCTACGGGAGGCAGCAG-3′) and 806R (5′-GGACTACHVGGGTWTCTAAT-3′) using an ABI GeneAmp® 9,700 PCR thermocycler (ABI, CA, USA) [22]. The PCR amplification of 16S rDNA gene was performed as follows: initial denaturation at 95°C for 3 min, followed by 55 cycles amplification (denaturation at 94°C for 15 s, primer annealing at 55°C for 15 s, and elongation at 72°C for 20 s) and single extension at 72°C for 10 min, and end at 4°C. The PCR mixtures contain 5 × TransStart FastPfu buffer 4 *μ*L, 2.5 mM dNTPs 2 *μ*L, forward primer (5 *μ*M) 0.8 *μ*L, reverse primer (5 *μ*M) 0.8 *μ*L, TransStart FastPfu DNA polymerase 0.4 *μ*L, template DNA 10 ng, and finally volume 20 *μ*L with ddH_2_O. PCR reactions were performed in triplicate. The PCR product was extracted from 2% agarose gel and purified using the AxyPrep DNA Gel Extraction Kit (Axygen Biosciences, Union City, CA, USA) according to manufacturer's instructions and quantified using Quantus™ Fluorometer (Promega, USA). Purified amplicons were pooled in equimolar and paired-end sequenced on an Illumina MiSeq PE300 platform/NovaSeq PE250 platform (Illumina, San Diego, USA) according to the standard protocols by Majorbio Bio-Pharm Technology Co., Ltd. (Shanghai, China).

The raw reads from 16S rDNA sequencing were demultiplexed, quality-filtered by fast version 0.20.0, and merged by FLASH version 1.2.7 [23, 24]. Operational taxonomic units (OTUs) with 97% similarity cutoff were clustered using UPARSE version 7.1, and chimeric sequences were identified and removed [25, 26]. The taxonomy of each OTU representative sequence was analyzed by RDP Classifier version 2.2 against the 16S rDNA database (e.g., Silva v138) using confidence threshold of 0.7 [27].

### 2.5. Transcriptome Sequencing

The transcriptome sequencing and analysis of colonic mucosa in piglets were completed by Majorbio Bio-Pharm Technology Co., Ltd. (Shanghai, China). The quality of transcriptome sequencing was reflected by the mapping ratio to the reference genome and transcriptome [28]. The expression was counted, and the read counts of each sample gene/transcript were obtained using RSEM and the results of alignment to the genome and genome annotation files [29]. Then they were transformed by fragments per kilobases per million reads (FPKM) or transcripts per million reads (TPM) to obtain a standardized gene/transcript expression. Differentially expressed genes (DEGs) were identified by DESeq2, DEGseq, and edgeR [30, 31, 32]. The difference of expression was analyzed, and DEGs were enriched by Gene Ontology (GO) and Kyoto Encyclopedia of Genes and Genomes (KEGG) pathway analysis.

### 2.6. Real-Time Quantitative PCR (RT-qPCR)

To verify the accuracy of transcriptome data, DEGs related to antibacterial and anti-inflammatory were selected for RT-qPCR verification. The total RNA of colonic mucosa was extracted with TRIzol™ reagent (Invitrogen Co., Ltd.), and then using electrophoresis on 1% agarose gel, optical density (OD) at 260 and 280 nm was measured [33]. The ratio of OD260 to OD280 of RNA in all samples was more than 1.8 [34]. After the concentration of the total RNA samples was standardized, the total RNA of each sample was reversely transcribed into the synthetic first strand (cDNA) with PrimeScript™ RT kit (TaKaRa, Dalian, China). Reverse transcription was completed in two steps; the genomic DNA reaction was first removed at 42°C for 2 min; and then transcription reaction was reversed at 37°C for 15 min and 85°C for 5 s. The reversely transcribed cDNA was stored at −80°C. Primers were compounded by Sangon (Shanghai, China) according to the gene sequence of pigs (see Table 2). The obtained cDNA was used as a template for the TB Green™ Premix Ex Taq™ PCR kit, which was used for RT-qPCR following the kit instructions (TaKaRa, Dalian, China). RT-qPCR conditions were as follows: 95°C incubation for 5 min followed by 40 cycles of denaturation at 95°C for 10 s and annealing at 64°C for 30 s, and the gene expression was calculated by 2^−*ΔΔ*CT^ method [35, 36]. In the process of reaction, the specificity of qPCR reaction and primers was detected according to melting curve analysis.

### 2.7. Statistical Analysis

Flora diversity and transcriptome data in colon were analyzed using software (http://www.majorbio.com). All the results were analyzed using SPSS 22.0 software for one-way ANOVA and Duncan's comparison. Data were expressed as mean ± SEM and drawn using GraphPad Prism 7.0. *P* < 0.05 means statistically significant.

## 3. Results

### 3.1. Effect of Quercetin on Growth Performance and Incidence of Diarrhea in Weaned Piglets

Compared with control, feed-to-gain ratio of the 500 and 750 mg/kg quercetin treatments was significantly reduced (*P* < 0.05); diarrhea rate and diarrhea index in the three quercetin treatments were significantly decreased in weaned piglets (*P* < 0.05); however, supplementation of dietary quercetin did not affect ADFI and ADG in weaned piglets (*P* > 0.05) (see Table 3).

### 3.2. Effect of Quercetin on Serum Parameters in Weaned Piglets

Compared with control, content of serum TGF-*β* in the three quercetin treatments was significantly increased (*P* < 0.05); content of serum NF-*κ*B in the 750 mg/kg quercetin treatment was significantly reduced (*P* < 0.05); content of serum IFN-*γ* in the 500 and 750 mg/kg quercetin treatments was significantly reduced (*P* < 0.05); however, content of serum TNF-*α* in the three quercetin treatments was not significantly different from that in control in weaned piglets (*P* > 0.05) (see Table 4).

### 3.3. Effect of Quercetin on Colonic Flora in Weaned Piglets

**Figure 1:**
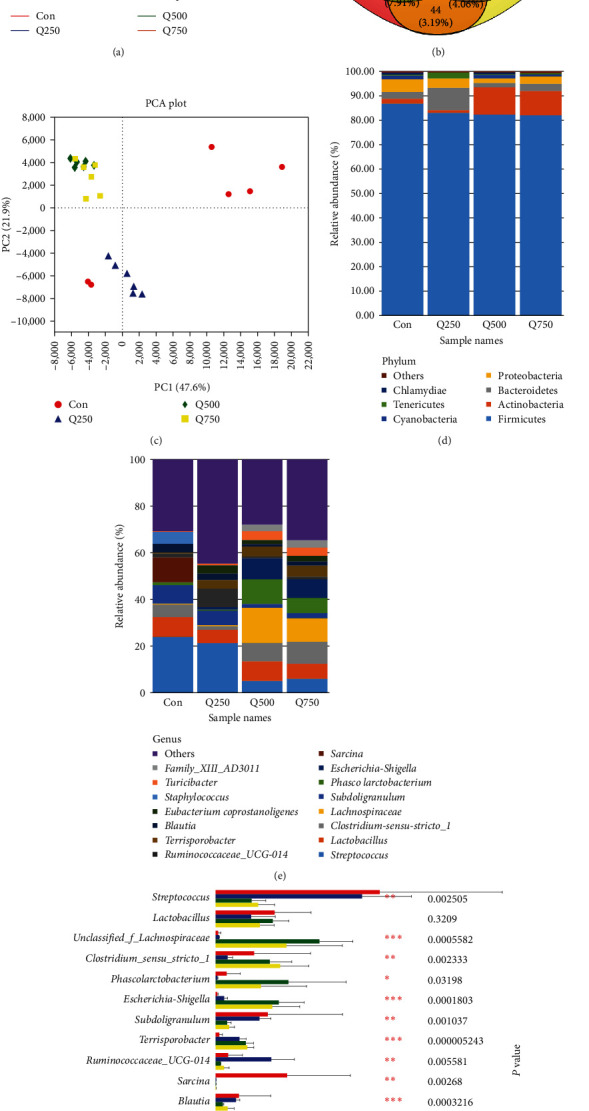
Description of flora community in the colon of weaned piglets: (a) rarefaction curves based on the observed taxonomic units (OTUs) in the colon, (b) Venn diagrams of shared genes, (c) principal component analysis (PCA) of flora in the colon, (d, e) the relative abundance of phyla and genera in the colon, and (f) the significant influence of genus level.  ^*∗*^,  ^*∗∗*^, and  ^*∗∗∗*^ represent *P* < 0.05, *n* = 6. Con represents control; Q250 represents 250 mg/kg quercetin treatment; Q500 represents 500 mg/kg quercetin treatment; and Q750 represents 750 mg/kg quercetin treatment.

The dilution curve gradually tended to smooth with increasing sequencing depth; it suggested that sequencing quantity was reasonable (see Figure 1(a)). The OTUs shared by the four treatments were 460, accounting for 33.38% of the total. There were 192, 96, 27, and 31 unique OTUs in control, 250, 500, and 750 mg/kg quercetin treatments (see Figure 1(b)), respectively. Principal component analysis showed that all samples in the same treatment were clustered together, indicating that the results were experimentally significant (see Figure 1(c)). However, location on coordinate axis of the 500 mg/kg quercetin treatment was close to the 750 mg/kg quercetin treatment, indicating that the microbial structures between the 500 and 750 mg/kg quercetin treatments were highly similar.

Compared with control, Ace index, Chao1 index, and Shannon index in the three quercetin treatments were significantly increased (*P* < 0.05); Sobs index was significantly increased in the 250 mg/kg quercetin treatment (*P* < 0.05); Simpson index in the 250 and 750 mg/kg quercetin treatments was significantly decreased in colonic contents of weaned piglets (*P* < 0.05) (see Table 5).

At the phylum level, composition of flora included Firmicutes, Proteobacteria, Bacteroidetes, Actinobacteria, and Cyanobacteria in colonic contents of weaned piglets (see Figure 1(d)). Firmicutes, Bacteroidetes, and Actinobacteria accounted for more than 90% and were the dominant bacteria in colonic contents of weaned piglets. Compared with control, the relative abundance of Actinobacteria in the 500 and 750 mg/kg quercetin treatments was significantly increased (*P* < 0.05) (see Figure 1(d)); the relative abundance of Proteobacteria in the three quercetin treatments was significantly decreased (*P* < 0.05) in colonic contents of weaned piglets (see Figure 1(d)).

At the genus level, the top 15 dominant genera were listed in all the treatments (see Figure 1(e)). Compared with control, the relative abundance of *Clostridium-sensu-stricto-1*, *unclassified_f_Lachnospiraceae*, *Phascolarctobacterium*, *Turicibacter*, and *Family_XIII _AD3011_group* was significantly increased (*P* < 0.05); the relative abundance of *Subdollgranulum* and *Blautia* was significantly decreased in the 500 and 750 mg/kg quercetin treatments (*P* < 0.05) (see Figure 1(f)); the relative abundance of *Escherichia-Shigella*, *Terrisporobacter*, and *Eubacterium-coprostanoligenes* was significantly increased (*P* < 0.05); the relative abundance of *Streptocococcus*, *Sarcina*, *Staphylococcus*, and *Ruminococcaceae_UCG-008* was significantly decreased in the three quercetin treatments (*P* < 0.05); the relative abundance of *Ruminococcaceae_UCG_014* in the 250 mg/kg quercetin treatment was significantly increased in colonic contents of weaned piglets (*P* < 0.05) (see Figure 1(f)).

### 3.4. Effect of Quercetin on Transcriptome in Colonic Mucosa of Weaned Piglets

Reads with low quality, joint contamination, and high unknown base N content were necessarily removed to ensure the reliability of the results. The original raw reads were filtered to get clean reads. The average clean reads of each sample were more than 93.75%; it suggested that the data of the samples were reliable (see Table 6). The Q20 of the clean reads data obtained from each sample sequencing was greater than 98.37%, and the Q30 was greater than 95.15%, indicating that the sequencing data met the requirements of subsequent biological information analysis (see Table 6).

**Figure 2:**
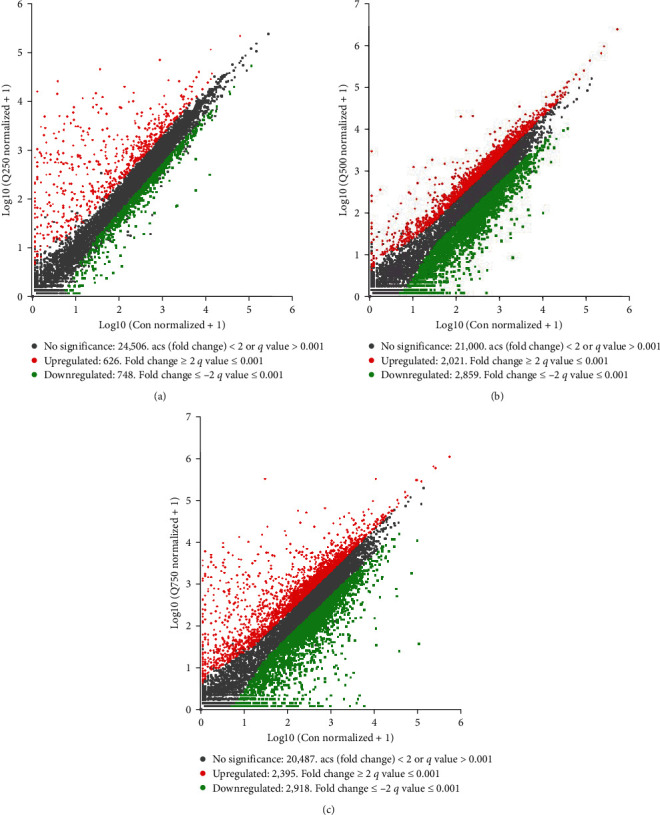
Effects of quercetin on differentially expressed genes in the colon of weaned piglets. The scatter plot showed the correlation of gene abundance. Red points represented the upregulated genes. Green points represented the downregulated genes, and gray dots represented significantly unchanged transcripts: (a) 250 mg/kg treatment, (b) 500 mg/kg treatment, and (c) 750 mg/kg treatment.

The analysis and screening of DEGs elucidated the sequencing of genes between control and the 250, 500, and 750 mg/kg quercetin treatments. Compared with control, 748 DGEs were significantly downregulated, and 626 DEGs were significantly upregulated in the 250 mg/kg quercetin treatment (see Figure 2(a)); 2,859 DGEs were significantly downregulated, and 2021 DEGs were significantly upregulated in the 500 mg/kg quercetin treatment (see Figure 2(b)); 2,918 DGEs were significantly downregulated, and 2,395 DEGs were significantly upregulated in the 750 mg/kg quercetin treatment (see Figure 2(c)).

**Figure 3:**
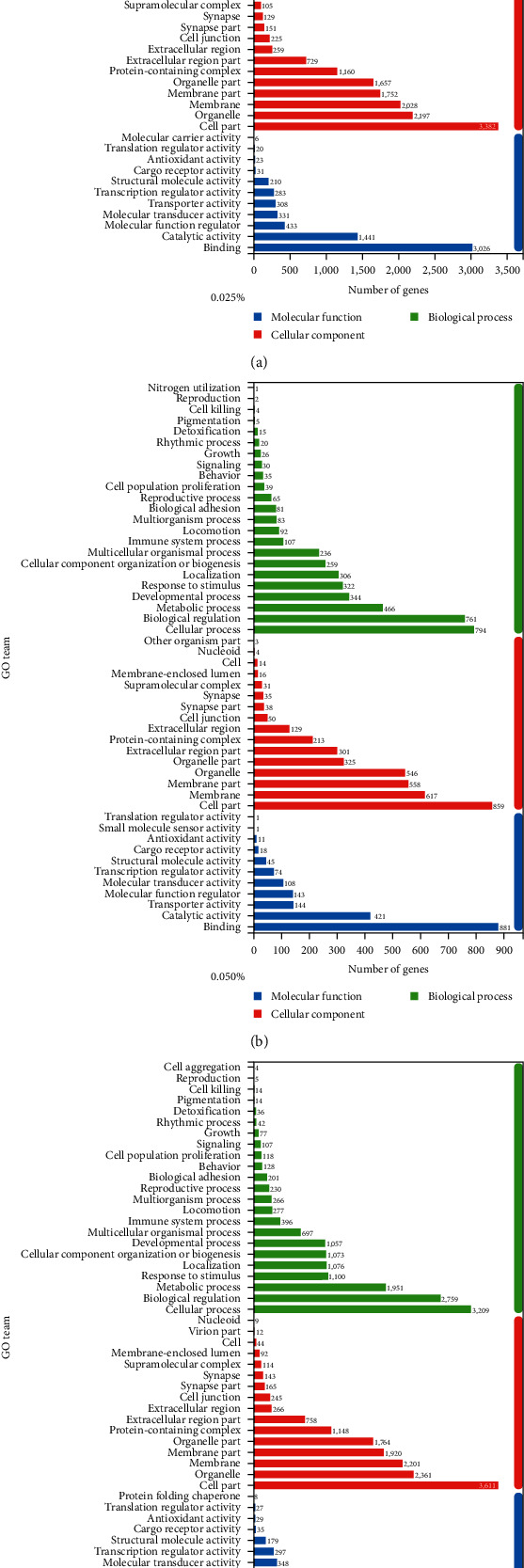
GO analyses of differentially expressed genes in control and 250 mg/kg quercetin treatment, 500 mg/kg quercetin treatment, and 750 mg/kg quercetin treatment. Figure 0.025% shown the GO analysis of control and 250 mg/kg quercetin treatment differentially expressed genes. Figure 0.05% shown the GO analysis of control and 500 mg/kg quercetin treatment differentially expressed genes. Figure 0.075% shown GO analysis of control and 750 mg/kg quercetin treatment differentially expressed genes: (a) 250 mg/kg quercetin treatment, (b) 500 mg/kg quercetin treatment, and (c) 750 mg/kg quercetin treatment.

GO enrichment and KEGG pathway were mainly involved in biological regulation, stimulation response, and cell and metabolic processes through the functional enrichment analysis (see Figure 3). Metabolic processes accounted for the third and immune system process accounted for the ninth in the most significantly enriched GO (see Figure 3). Numerous DEGs were associated with function of inflammatory cytokines and metabolic processes after GO annotation of these DEGs. KEGG biological pathway classification and enrichment analysis on DEGs showed that 303, 328, and 332 enrichment pathways were observed in the 250, 500, and 750 mg/kg quercetin treatments, respectively, compared with control. Enriched pathways related to antibacterial and anti-inflammatory activity were screened out (see Table 7). Phosphoinositol 3kinase-seronine protein kinase (PI3K–Akt) signaling pathway, MAPK signaling pathway, and NF-*κ*B signaling pathway were significantly affected by quercetin treatments (*P* < 0.05).

### 3.5. Validation of NF-*κ*B Signaling Pathway by RT-qPCR

**Figure 4:**
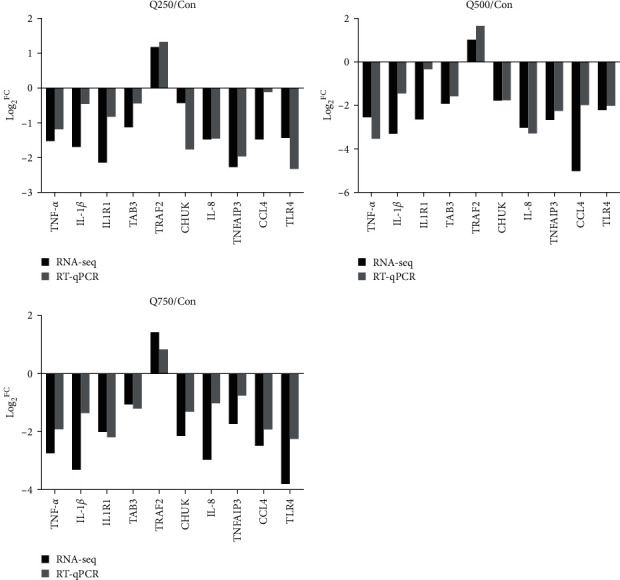
Correlation of mRNA expression of 10 random DEG sequences of anti-inflammatory factors in NF-*κ*B pathway using RNA-seq and RT-qPCR. The three diagrams shown the results of comparing Q250, Q500, and Q750 with Con. Black represented the RNA-seq result, and gray represented the RT-qPCR result. Con represented control; Q250 represented 250 mg/kg quercetin treatment; Q500 represented 500 mg/kg quercetin treatment; and Q750 represented 750 mg/kg quercetin treatment.

**Figure 5:**
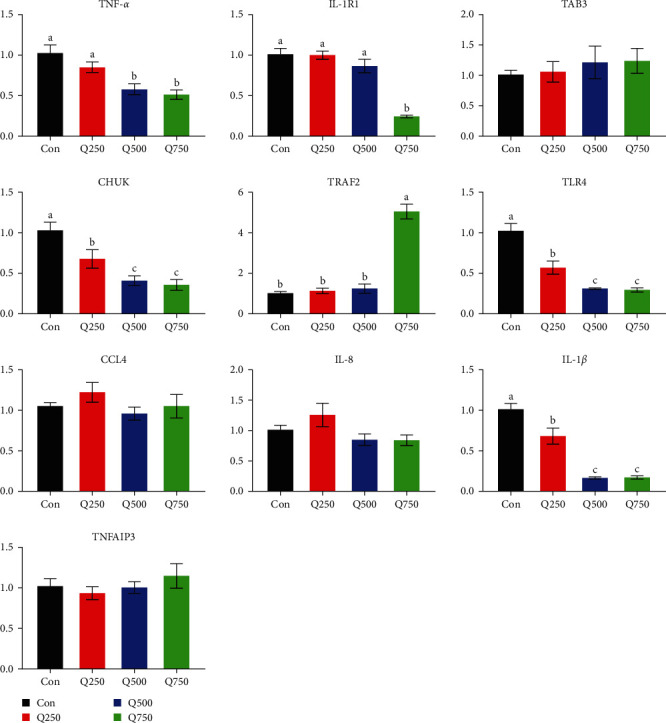
The expression of 10 DEGs was detected using RT-qPCR. Mean values without a common letter are significantly different, *P* < 0.05. Values were mean ± SEM, *n* = 6. Con represents control; Q250 represents 250 mg/kg quercetin treatment; Q500 represents 500 mg/kg quercetin treatment; and Q750 represents 750 mg/kg quercetin treatment.

To validate the accuracy of RNA-seq results, 10 genes from NF-*κ*B signaling pathway were randomly selected, including one of significantly upregulated genes (TRAF2) and nine of significantly downregulated genes (TLR4, chemokine (C–C motif), ligand 4 (CCL4), tumor necrosis factor alpha-induced protein 3 (TNFAIP3), interleukin-8 (IL-8), CHUK, TAK1-binding protein 3 (TAB3), IL1R1, IL-1*β*, and TNF-*α*) (see Table 8). The present results showed that gene expression based on RT-qPCR and RNA-seq was similar, indicating that RNA-seq data were credible and accurate (see Figure 4). Compared with control, mRNA expression of TNF-*α*, IL1R1, CHUK, TLR4, and IL-1*β* were significantly decreased with increasing quercetin (*P* < 0.05), and the expression of TRAF2 mRNA in the 750 mg/kg quercetin treatment was significantly increased (*P* < 0.05) (see Figure 5).

## 4. Discussion

Lack of necessary immunoenhancing substances in breast milk seriously affects growth of postweaned piglets; meanwhile, weaning stress may result in intestine dysfunction, inflammation, and gut microbiota disruption, ultimately augmenting diarrhea [20, 37, 38]. Therefore, some additives have been applied in improving the weaning stress of piglets. Many studies have shown that flavonoids improved performance and intestinal barrier of animals [6]. Supplementation of dietary garcinol effectively increased growth performance and decreased diarrhea through enhancing antioxidant capacity and changing intestinal flora, thus improving intestinal dysfunction and inflammation in 144 weaned piglets at 28 days of age [39]. A certain dose of grape seed procyanidins increased ADG, decreased feed-to-gain ratio, and incidence of diarrhea in 96 piglets at 28 days of age [40]. Diet supplemented with 300 ppm laminarin-rich extract significantly increased ADG and ADFI, thereby improving performance and preventing intestinal dysfunction in 96 postweaned piglets for 14 days [41]. The diet supplemented with 0–300 g/t mulberry leaf extract (rich in alkaloids and flavonoids) significantly increased the feed conversion rate and reduced the diarrhea rate in 30 weaned piglets for 42 days [42]. Liu et al. [2] proved that quercetin improved feed conversion, thereby improving egg production performance in two hundred forty 28-week-old laying hens. Similarly, Goliomytis et al. [43] found that quercetin significantly increased feed conversion rate of broilers for 42 days. Quercetin tended to increase feed conversion rate in pigs for 7 weeks [44]. The diet supplemented with 50–200 mg/kg guava leaf extract reduced *Escherichia* coli-induced diarrhea, regulated immune response, and maintained intestinal health in weaned piglets [45]. Our experiment showed that quercetin significantly reduced feed to gain rate and incidence of diarrhea, thereby improving growth performance in weaned piglets; this further verified growth promoting action of quercetin, like the above-mentioned flavonoids.

To further clarify the mechanism of quercetin influencing growth performance and incidence of diarrhea in weaned piglets, the serum indicators, colonic flora, and transcriptome were determined. The metabolism and health may be reflected by serum indexes in weaned piglets. Cytokines were important mediators of anti-inflammation and proinflammation in animals. Stimuli from various stressors increased local or systemic proinflammatory cytokines and resulted in an imbalance between proinflammatory and anti-inflammatory responses, thereby inducing inflammation which may interfere with normal intestinal integrity in animals [46, 47, 48, 49]. Factors inducing the inflammatory cascade include TNF-*α*, chemokines, IFN-*γ*, etc. Anti-inflammatory cytokines, including IL-10 and TGF-*β*, may inhibit inflammation by regulating proinflammatory cytokine reaction [50]. Flavonoids play an anti-inflammatory role by regulating expression and activation of cytokines including interleukin and tumor necrosis factor and inhibiting expression of proinflammatory factors NF-*κ*B [51]. Clinical trials have shown that quercetin supplementation significantly improved clinical symptoms and plasma TNF-*α* levels in patients with rheumatoid arthritis [52]. Besides, chrysin reduced the levels of serum IFN-*γ* and TNF-*α* in mice for 8 days [53]. Total flavonoids from *Prunella vulgaris* increased the expression of TNF-*α* and IFN-*γ* in serum of tumor-bearing mice and improved anticancer and anti-inflammatory ability in SMMC-7721 cells [54]. Our experiment found that quercetin significantly increased the content of serum TGF-*β* and significantly decreased the content of serum NF-*κ*B and IFN-*γ*. This further confirmed the anti-inflammatory effect of quercetin. However, quercetin did not significantly affect content of serum TNF-*α* in weaned piglets of this present study; it probably resulted from difference of the status of body (diseases or health), study subjects, and types of flavonoids.

Clearly, the optimal growth gastrointestinal tract (GIT) is important to the overall physiological metabolism and disease state in pigs at all stages of growth and development [37]. There are many physiological and gastrointestinal factors from weaning transition in influencing postweaned diarrhea, one key of which is microbiota disruption in the GIT [37]. Therefore, it is very important to maintain the balance of intestinal microbiota in piglets. The higher the diversity of the flora in animal's intestines, the more stable and balanced the intestinal microecology [55]. Ketones and polyphenols may affect the composition of intestinal flora, including increase in beneficial bacteria, decrease in harmful bacteria, and regulate the steady state of microorganisms in intestine, thus promoting the digestion and absorption of nutrients, ultimately improving the body's health, growth, and development [56]. The previous study of our team showed that quercetin improved production performance through regulating microflora in laying hens during the late laying period [57]. Hence, the colonic flora was studied using 16S rDNA sequencing technique to clarify the relationship between flora and diarrhea in weaned piglets in the present study. We found that Firmicutes, Bacteroidetes, and Actinobacteria were the dominant flora in colon of weaned piglets, which was consistent with previous studies [9]. In the present study, the relative abundance of Actinobacteria was significantly increased in the 500 and 750 mg/kg quercetin treatments, and the relative abundance of Proteobacteria was significantly decreased in the three quercetin treatments. The function of Actinobacteria powerfully enhances animal body's immunity because it may generate metabolites with a variety of biological functions, including antibacterial drugs, immunosuppressive agents, antioxidants, and plant growth hormones [58]. Proteobacteria is a large class of bacteria, including *Escherichia coli*, Salmonella, *Vibrio cholerae*, and *Helicobacter pylori*. If these pathogens were increased, the disorder of intestinal flora was disturbed, and immunity was decreased, thus increasing the occurrence of diarrhea [59]. Study of Mirpuri et al. [60] highlighted the importance of IgA in establishing a beneficial commensal bacterial population via selectively suppressing Proteobacteria needed for reducing susceptibility to colonic injury and inflammation. Shin et al. [61] found that population of Proteobacteria in the intestinal tract of healthy people was low; however, it was significantly high in patients with diarrhea, accompanied by increasing proinflammatory cytokines. Lachnospiraceae is an important microorganism in the intestine of pigs, may degrade fiber, and promote the decomposition of cellulose. Fiber and cellulose are not easy to digest into monosaccharides and short-chain fatty acids and absorb; however, products of fiber and cellulose degradated by Lachnospiraceae are easy to absorb and utilize, thereby promoting energy utilization in animals [62]. The abundance of Lachnospiraceae was significantly increased in gut microbiome, while the abundance of *Streptococcus* was significantly decreased; Lachnospiraceae also produced short-chain fatty acids and butyrate in the intestine to regulate intestinal homeostasis in clinical trials of 40 participants consumed (serving a daily 380 mL) flavonoid-rich orange juice for 8 weeks [63]. In addition, the diet supplemented with 0.1% quercetin significantly increased the abundance of Lachnospiraceae, and the diversity of flora and shaped the specific flora structure in intestine of 50 obese rats for 12 weeks [64]. *Eubacterium limosum* may metabolize flavonoids with a methoxy group, such as isoxanthohumol and icaritin [65]. *Sarcina* was one part of the flora in animal and human skin and large intestine; however, it may induce gastrointestinal swelling and is a pathogenic bacterium in animals [66]. In the present study, quercetin significantly decreased the abundance of *Sarcina*, and the abundance of Lachnospiraceae was significantly increased in weaned piglets. Studies have shown that quercetin may release reactive oxygen species on the biofilm surface of strains including *Staphylococcus aureus*, *E. coli*, and *Pseudomonas aeruginosa*, thus resulting in biofilm destruction and microbial cell death; therefore, we suspect that the antibacterial mechanism of quercetin in the gut was the same as the study [67]. The present study indicated that quercetin regulated the dynamic balance of beneficial and harmful bacteria, improved function and distribution of intestinal flora, along with the decreased proinflammatory cytokines, and thus promoted health in the intestines.

Besides, the colonic mucosa was analyzed using transcriptome sequencing to further understand the mechanism of quercetin influencing growth performance and diarrhea in weaned piglets. The biological significance may be mined from a large number of gene expression data through bioinformatics with rapidly developed sequencing technology, thereby making up for the shortcomings of traditional biology. Studies on animal transcriptome may reveal the molecular mechanisms underlying animal stressors and cellular changes [68]. Some studies have shown that quercetin regulated inflammation and immune response [69]. In our experiment, 25,880 DEGs and a variety of signal pathways were obtained from the sequencing data of quercetin treatments, involving in the antibacterial and anti-inflammatory process in colonic mucosa of weaned piglets.

The functional distribution of the DEGs was analyzed in the colonic mucosa of weaned piglets supplemented with dietary quercetin (250, 500, and 750 mg/kg quercetin) and compared with control using GO functional classification and KEGG pathway analysis to understood the regulatory network of antibacterial and anti-inflammatory pathways. GO may directly analyze the overall function of the whole gene sets, and the DEGs are divided into three parts, including molecular function, cellular component, and biological process. In the present study, GO analysis showed that many DGEs were related to cytokines and immune response, including TLR4, CCL4, TNFAIP3, CXCL8, CHUK, TRAF2, TAB3, IL1R1, IL1*β*, and TNF-*α*, and these DEGs were associated with inflammation. Inflammatory cytokines play an important role in regulating immune and inflammatory response and intestinal integrity and are closely associated with the occurrence of diarrhea [70]. Meanwhile, some DEGs in NF-*κ*B signaling pathway were selected for RT-qPCR to prove the accuracy of RNA-seq results. The results showed that the results of RNA-seq were highly consistent with those of RT-qPCR.

In the present study, KEGG pathway analysis showed that many significant DGEs were related to immune response and inflammatory pathways, and 205 DGEs were enriched in NF-*κ*B signaling pathway. NF-*κ*B signaling pathway plays a key role in regulating gene expression induced by cytokines [71]. It also participates in many biological processes, including immune response, inflammatory response, apoptosis, and tumorigenesis [72]. A variety of proinflammatory factors may activate NF-*κ*B signaling pathway through inhibiting IKK-dependent phosphorylation and ubiquitin-mediated I*κ*B protein degradation in animals [73]. IKK*α* (also known as CHUK) was an important regulator of nonclassical NF-*κ*B pathway. Flavonoids may control inflammation through inhibiting NF-*κ*B signaling pathway. Quercetin may also induce apoptosis by inhibiting NF-*κ*B signaling pathway in human colon cancer cells [73]. In addition, quercetin may inhibit TNF-*α*-induced inflammation and apoptosis via blocking NF-*κ*B signal pathway in endothelial cells [18].

In this study, mRNA expression of TNF-*α*, IL1R1, CHUK, TLR4, and IL-1*β* was decreased in a dose-dependent manner with increasing quercetin using RT-qPCR in colonic mucosa of weaned piglets. TNF-*α* and IL-1*β* were the main proinflammatory factors in organism, activating NF-*κ*B and mediating inflammatory response. Inhibiting NF-*κ*B may play a role in anti-inflammation. IL-1 receptor 1 exists on the surface of almost all nucleated cells and transmits activation signals; its expression was positively consistent with IL-1*β* [74]. Some studies have shown that 10 *μ*M quercetin significantly reduced production of TNF-*α* and IL-1*β* in the trachea and alveolar cells [75]. Quercetin also inhibited TNF-*α*-induced apoptosis and inflammatory response by blocking NF-*κ*B and AP-1 signaling pathway in human umbilical vein endothelial cells [18]. In addition, quercetin (25–100 *μ*M) inhibited mRNA expression of TNF-*α* and IL-1*β*, thus reducing inflammation in human peripheral blood mononuclear cells [76]. Ding et al. [77] found that 200 *μ*M quercetin inhibited expression of IL-4/TNF-*α* mRNA in conjunctival tissue and inflammation in mouse model. We also found that quercetin inhibited mRNA expression of TNF-*α* and IL-1*β*, which was consistent with the above-mentioned studies. TLRs are complete transmembrane glycoprotein, which play an important role in cellular immunity and inflammatory signal transduction. TLR4 was an innate immune system pathogen pattern recognition receptor and may also act as a lipopolysaccharide recognition receptor. It locates in the upstream stage of inflammatory signal transduction and is the first link in regulating inflammatory response [78]. Generally, TLR4 expression is low in intestinal epithelium of healthy mammals. Some studies have confirmed that TLR4 expression was significantly increased under various stimuli, such as ulcerative colitis [79]. Bhaskar et al. [80] found that quercetin inhibited the ox-LDL-induced mRNA expression of TLR2 and TLR4 and regulated the TLR-NF-*κ*B signaling pathway and thereby inhibited the cytokine production and downregulated the activity of inflammatory enzymes. Fifty milligram per kilogram quercetin inhibited expression of inflammatory cytokines and TLR4/NF-*κ*B signal in mouse cells [81, 82], which was consistent with our results that TLR4 expression was significantly downregulated. Together with the above-mentioned results from cytokines, quercetin inhibited inflammation and reduced stress through inhibiting mRNA expression of TLR4, IL-1*β*, CHUK, and TNF-*α* in colonic mucosa of weaned piglets. Importantly, quercetin excerted significant growth-promoting and antidiarrhea effect in this experiment. Meanwhile, quercetin acts as a natural anti-inflammatory and antibacterial component with wide source and no-drug resistance in animals. Therefore, quercetin presented great potential in applying for animal husbandry as a functional feed additive.

## 5. Conclusions

Quercetin increased the feed conversion rate and reduced the incidence of diarrhea through regulating flora and the NF-*κ*B signaling pathway in the colon of weaned piglets.

## Figures and Tables

**Table 1:** Composition and nutrient levels of the basal diet (dry matter basis).

Raw material	Content (%)	Nutrient levels (%)	
Corn	62.07	DE (MJ/kg) ^*∗∗*^	14.82
Full-fat soybean	9.06	Crude protein (CP)	18.98
Peeled soybean meal	13.00	Phosphorus	0.60
Corn gluten meal	2.51	Calcium	0.71
Soybean oil	2.84	Lys	1.23
Fish meal	2.90	—	—
Whey powder	4.00	—	—
Calcium hydrogen phosphate	1.16	—	—
Limestone	0.65	—	—
Sodium chloride	0.45	—	—
Lysine	0.33	—	—
Methionine	0.03	—	—
Premix ^*∗*^	1.00	—	—
Total	100.00	—	—

^*∗*^*Note*: The diet per kg contains vitamin A, 5,000 IU; vitamin D3, 1,000 IU; vitamin E, 40 mg; vitamin K3, 1 mg; vitamin B1, 1 mg; vitamin B2, 4 mg; vitamin B6, 2.5 mg; vitamin B12, 0.02 mg; niacin, 10 mg; pantothenic acid, 12 mg; folic acid, 1.2 mg; biotin, 0.2 mg; iron (FeSO_4_ · 7H_2_O), 200 mg; copper (CuSO_4_ · 5H_2_O), 25 mg; zinc (ZnSO_4_ · 7H_2_O), 80 mg; manganese (MnSO_4_ · H_2_O), 130 mg; selenium (NaSeO_3_), 0.5 mg; iodine (KI), 1 mg.  ^*∗∗*^Nutrient levels were calculated.

**Table 2:** Primers of genes related to NF-*κ*B signaling pathway used for mRNA expression.

Genes	Primer	Sequence (5′→3′)	Prod size
TLR4	F	AGGACGAAGACTGGGTGAGGAATG	126
	R	CCTGGATGATGTTAGCAGCGATGG	
CCL4	F	TGGTCCTGGTCGCTGCCTTC	94
	R	TTCCGCACGGTGTATGTGAAGC	
TNFAIP3	F	ACAATGAGCAGGGGCGGAGAG	150
	R	CTGAGCACTCGTGGCACAAGG	
CXCL8	F	TCCAAACTGGCTGTTGCCTTCTTG	132
	R	GGGGTGGAAAGGTGTGGAATGC	
CHUK	F	GCGTCAGGGAGACTTGATGGAATC	101
	R	CCGTGCTGTCGCTGTAAGAGTG	
TRAF2	F	AACATCGTCTGCGTGCTGAACC	114
	R	CACCTTGTTGCTCAGGGCTTCG	
TAB3	F	CCACAGCTCGATATGCAGGTTCTC	120
	R	GGCTCGGCAACAGGCTTCAAG	
IL1R1	F	CCGCACACAATGGGCACTATACC	146
	R	CATCGTCTCATTGGCTGGACTGAC	
IL1B	F	GCCAACGTGCAGTCTATGGAGTG	91
	R	GGTGGAGAGCCTTCAGCATGTG	
TNF	F	GCACTGAGAGCATGATCCGAGAC	120
	R	CGACCAGGAGGAAGGAGAAGAGG	
*β*-Actin	F	TCTGGCACCACACCTTCT	114
	R	TGATCTGGGTCATCTTCTCAC	

**Table 3:** Effect of quercetin on growth performance and incidence of diarrhea in weaned piglets.

Items	Control	Quercetin (mg/kg)			SEM	*P* value
		250	500	750		
ADG (g)	433.33	435.00	439.17	468.33	12.40	0.73
ADFI (g)	710.13	710.52	693.33	736.66	7.70	0.27
F/G	1.65^a^	1.63^a^	1.55^b^	1.54^b^	0.05	<0.05
Diarrhea rate	15.24^a^	9.28^b^	5.17^c^	4.53^c^	1.31	<0.05
Diarrhea index	1.16^a^	1.10^b^	1.07^c^	1.05^c^	0.01	<0.05

*Note*: Statistical analysis was performed by one-way ANOVA followed by Duncan's test. Values are expressed as means ± SEM, and *n* = 6. Values with different letters are significantly different (*P* < 0.05). Values with no letter or the same letter superscripts mean no significant difference (*P* > 0.05).

**Table 4:** Effect of quercetin on serum inflammatory factors in weaned piglets.

Item	Control	Quercetin (mg/kg)			SEM	*P* value
		250	500	750		
NF-*κ*B (pg/mL)	818.38^b^	814.50^b^	852.38^ab^	712.93^c^	20.54	0.01
TGF-*β* (pg/mL)	237.43^c^	309.94^b^	506.04^a^	484.61^a^	24.62	<0.05
TNF-*α* (pg/mL)	72.72	71.33	65.11	78.95	3.04	0.48
IFN-*γ* (pg/mL)	720.78^a^	729.09^a^	547.75^b^	419.20^c^	28.32	<0.05

*Note*: Statistical analysis was performed by one-way ANOVA followed by Duncan's test. Values are expressed as means ± SEM, and *n* = 6. Values with different letters are significantly different (*P* < 0.05). Values with no letter or the same letter superscripts mean no significant difference (*P* > 0.05).

**Table 5:** Effect of quercetin on the alpha diversity of colonic flora in weaned piglets.

Item	Control	Quercetin (mg/kg)			SEM	*P* value
		250	500	750		
Sobs	479.50^b^	633.83^a^	513.67^b^	543.33^b^	15.79	0.01
Ace	584.54^b^	741.18^a^	677.32^a^	717.27^a^	18.47	<0.05
Chao 1	580.66^c^	760.19^a^	678.18^b^	719.67^ab^	18.49	<0.05
Simpson	0.09^a^	0.05^b^	0.07^ab^	0.05^b^	0.01	0.01
Shannon	3.28^c^	4.38^a^	3.72^b^	3.97^b^	0.10	<0.05

*Note*: Statistical analysis was performed by one-way ANOVA followed by Duncan's test. Values are expressed as means ± SEM, and *n* = 6. Values with different letters are significantly different (*P* < 0.05). Values with no letter or the same letter superscripts mean no significant difference (*P* > 0.05).

**Table 6:** Summary of sequence quality and alignment information of colonic mucosa samples from weaned piglets.

Item	Control	Quercetin (mg/kg)		
		250	500	750
Clean reads	55,622,022.00	51,638,123.00	50,400,244.00	47,705,921.00
Q20 (%)	98.45	98.51	98.47	98.37
Q30 (%)	95.25	95.38	95.33	95.15
Total mapped reads	51,732,595 (92.87%)	49,012,669 (94.91%)	47,585,568 (94.41%)	44,246,702 (92.83%)
Multiple mapped reads	2,487,822 (4.47%)	2,157,064 (4.19%)	3,059,086 (5.99%)	2,919,126 (6.08%)
Uniquely mapped reads	49,244,772 (88.40%)	46,855,605 (90.72%)	44,526,482 (88.42%)	4,327,576 (86.75%)

*Note*: Clean reads, statistics of the number of sequenced sequences after filtering; total mapped, the number of clean reads mapped to the genome; multiple mapped, the number of clean reads with multiple alignment positions on the reference sequence; unique mapped, the number of clean reads with unique alignment on the reference sequence. *n* = 6.

**Table 7:** Quercetin regulates antibacterial and anti-inflammatory pathways in the colon of weaned piglets.

Pathway ID	Pathway definition	*P* value			All genes
		Q250/Con	Q500/Con	Q750/Con	
map04933	AGE–RAGE signaling pathway in diabetic complications	2.57E−05	7.78E−07	724E−03	118
map04151	PI3K–Akt signaling pathway	1.28E−09	2.93E−11	6.03E−05	464
map04064	NF-*κ*B signaling pathway	1.71E−02	5.35E−13	4.20E−05	305
map04666	Fc gamma R-mediated phagocytosis	4.24E−01	5.66E−07	1.20E−03	150
map04144	Endocytosis	9.71E−01	3.88E−01	1.45E−01	272
map04668	TNF signaling pathway	3.27E−01	1.88E−03	4.28E−04	120
map04010	MAPK signaling pathway	1.14E−03	1.12E−03	4.70E−03	322
map05218	Melanoma	3.73E−03	1.53E−01	2.31E−01	82
map04916	Melanogenesis	9.29E−02	2.13E−01	9.50E−02	116
map00590	Arachidonic acid metabolism	3.68E-02	1.74E-01	1.34E-02	75
map04621	NOD-like receptor signaling pathway	3.67E−02	1.72E−02	3.66E−04	175
map04722	Neurotrophin signaling pathway	9.88E−01	3.28E−01	3.11E−01	132
map04630	Jak-STAT signaling pathway	0.082814	3.34E−03	1.05E−01	181
map05321	Inflammatory bowel disease (IBD)	7.76E−01	1.67E−09	2.63E−01	125
map04620	Toll-like receptor signaling pathway	7.77E−02	4.87E−02	1.89E−01	112
map04350	TGF-*β* signaling pathway	2.65E−02	2.06E−01	3.95E−01	94

*Note*: *P* value, significant statistical value. Con represents control; Q250 represents 250 mg/kg quercetin treatment; Q500 represents 500 mg/kg quercetin treatment; and Q750 represents 750 mg/kg quercetin treatment.

**Table 8:** Differentially expressed genes enriched in NF-*κ*B signaling pathway.

Gene ID	Gene name	Log_2_^FC^			Type
		Q250/Con	Q500/Con	Q750/Con	
ENSSSCG00000001404	TNF-*α*	−1.52	−2.54	−2.75	NF-*κ*B signaling pathway
ENSSSCG00000039214	IL-1*β*	−1.69	−3.30	−3.31	NF-*κ*B signaling pathway
ENSSSCG00000008162	IL1R1	−2.14	−2.65	−2.01	NF-*κ*B signaling pathway
ENSSSCG00000012203	TAB3	−1.13	−1.93	−1.06	NF-*κ*B signaling pathway
ENSSSCG00000005838	TRAF2	1.18	1.02	1.43	NF-*κ*B signaling pathway
ENSSSCG00000010548	CHUK (IKK*α*)	—	−1.78	−2.15	NF-*κ*B signaling pathway
ENSSSCG00000008953	CXCL8 (IL-8)	−1.47	−3.02	−2.97	NF-*κ*B signaling pathway
ENSSSCG00000004154	TNFAIP3 (A20)	−2.27	−2.66	−1.74	NF-*κ*B signaling pathway
ENSSSCG00000038096	CCL4 (MIP1*β*)	−1.47	−5.02	−2.49	NF-*κ*B signaling pathway
ENSSSCG00000005503	TLR4	−1.43	−2.21	−3.8	NF-*κ*B signaling pathway

*Note*: FC (s1/s2), the difference ratio of the gene/transcript between the two samples; log_2_^FC (s1/s2)^, the logarithm with base 2 as the difference ratio between the two samples for the gene/transcript and s2 as the control. Con represents control; Q250 represents 250 mg/kg quercetin treatment; Q500 represents 500 mg/kg quercetin treatment; and Q750 represents 750 mg/kg quercetin treatment.

## Data Availability

The data used to support the findings of this study are available from the corresponding author upon request.
